# AC conductivity and correlation effects in nano-granular Pt/C

**DOI:** 10.1038/s41598-021-94575-w

**Published:** 2021-07-26

**Authors:** Marc Hanefeld, Peter Gruszka, Michael Huth

**Affiliations:** Physikalisches Institut, Goethe Universität, Frankfurt am Main, 60438 Germany

**Keywords:** Nanoscale materials, Materials science, Electronic properties and materials

## Abstract

Nano-granular metals are materials that fall into the general class of granular electronic systems in which the interplay of electronic correlations, disorder and finite size effects can be studied. The charge transport in nano-granular metals is dominated by thermally-assisted, sequential and correlated tunneling over a temperature-dependent number of metallic grains. Here we study the frequency-dependent conductivity (AC conductivity) of nano-granular Platinum with Pt nano-grains embedded into amorphous carbon (C). We focus on the transport regime on the insulating side of the insulator metal transition reflected by a set of samples covering a range of tunnel-coupling strengths. In this transport regime polarization contributions to the AC conductivity are small and correlation effects in the transport of free charges are expected to be particularly pronounced. We find a universal behavior in the frequency dependence that can be traced back to the temperature-dependent zero-frequency conductivity (DC conductivity) of Pt/C within a simple lumped-circuit analysis. Our results are in contradistinction to previous work on nano-granular Pd/$\hbox {ZrO}_2$ in the very weak coupling regime where polarization contributions to the AC conductivity dominated. We describe possible future applications of nano-granular metals in proximity impedance spectroscopy of dielectric materials.

## Introduction

Alternating current (AC) charge transport in disordered materials has been an intensively researched topic over many years, see e.g.^1,2^. From this research, covering a broad range of materials classes of both, ionic and electronic conductors, a universal AC conductivity behavior has been deduced. This is most prominently reflected by a sub-linear power law behavior of the real part of the AC conductivity observed above a temperature-dependent characteristic frequency, whereas the conductivity is frequency-independent at lower frequencies^1^. Interestingly, nano-granular metals^3^, being a prominent example of electronically conducting disordered materials, have been investigated only very recently with regard to their AC conductivity behavior^4,5^. Indeed, for Pd nanoparticles embedded into $\hbox {ZrO}_2$ a power law behavior of the real part of the AC conductivity was found in the temperature range from about 40 to 100 K with an exponent decreasing from 0.71 to 0.55 with increasing temperature^5^. This observation was made on highly resistive samples and at this point it is important to briefly recapitulate that two components contribute to the AC conductivity of electronic conductors in the linear regime, as follows

1 $$\begin{aligned} \sigma (\omega ) = \sigma _f(\omega ) - i\omega \varepsilon _0\varepsilon _r(\omega ) \end{aligned}$$

where the first part reflects the complex conductivity contribution of free charges and the second part stands for polarization effects characterized by the frequency-dependent dielectric function. For a nano-granular metal the free-charges contribution stems from the electrons tunneling between the metallic nanoparticles. Assuming for simplicity a Debye-like polarization contribution of the form

2 $$\begin{aligned} \varepsilon _r(\omega ) = \varepsilon _\infty + \frac{\varepsilon _r(0) - \varepsilon _\infty }{1 + i\omega \tau } \end{aligned}$$

with high-frequency limit $\varepsilon _\infty$ and relaxation time $\tau$, it follows

3 $$\begin{aligned} \sigma (\omega ) = \sigma _f(\omega ) - i\omega \varepsilon _0\left( \varepsilon _\infty + \frac{\varepsilon _r(0) - \varepsilon _\infty }{1 + i\omega \tau } \right) \end{aligned}$$

from which an order of magnitude estimation regarding the two conductivity contributions can be deduced

$$\begin{aligned} {{\mathcal {O}}}(\left| \sigma \right| ) = {{\mathcal {O}}}(\left| \sigma _f \right| ) + {{\mathcal {O}}}(\omega )\times 10^{-11} \end{aligned}$$

whereby realistically assuming ${{\mathcal {O}}}(\left| \varepsilon _\infty \right| ) = {{\mathcal {O}}}(\left| \varepsilon _r(0) \right| ) = 1$. For the Pd/$\hbox {ZrO}_2$ samples studied in^5^ inspection of the conductivity data shows an upper limit for $\sigma (0) \equiv \sigma _f(0)$ of $10^{-12}\,$S/m. From this we conclude that already at frequencies above 10 Hz the polarization contribution to the AC conductivity by far dominated the $\sigma _f(\omega )$ part. The observed universal frequency dependence of the AC conductivity therefore reflects the polarization contributions. This is an important observation with regard to the question whether electronic correlation effects influence the AC conductivity behavior of nano-granular metals, i.e. in the case that the charging energy of the nanoparticles is the dominating energy scale in the relevant tunneling processes^6,7^. The results obtained for Pd/$\hbox {ZrO}_2$ cannot shade light on this question which is the main focus of this work. To gain a deeper insight into the influence of correlation effects onto the AC-response of nano-granular metals is not only a matter of basic research on the electronic properties of this material class close to the insulator-metal transition. There are in fact foreseeable applications of nano-granular metals in dielectric proximity sensing, i.e. the detection of changes in the dielectric medium in close proximity to the nano-granular metal by way of their influence on the electronic correlation effects^8,9^.

Here we present results on the AC conductivity of the nano-granular metal Pt/C, i.e. Pt nanoparticles embedded into amorphous carbon, fabricated by the direct-write process of focused electron beam induced deposition (FEBID); see^10–12^ for recent reviews on FEBID. Importantly, the inter-granular tunnel coupling strength between the Pt nanoparticles can be finely tuned by post-growth electron irradiation of the samples to such an extent that an increase of four orders of magnitude of the conductivity can be obtained driving the samples from the insulating side through a insulator-metal transition into the quasi-metallic regime^13,14^. In the work presented here we focus on a set of samples covering the insulating side of the phase diagram with room temperature DC conductivities $\sigma (0) = \sigma _f(0)$ of 10 S/m or above. Therefore, the frequency-dependent conductivity is by far dominated by the free-charge contribution which allows us to study the influence of electronic correlation effects. In particular, we demonstrate within a quantitative lumped-circuit model approach that the correlated variable-range hopping (cVRH) temperature dependence found for the DC conductivity of all samples is fully reflected in the AC conductivity. From the deduced temperature dependence of the model parameters we find evidence for a small non-percolating metallic conductivity contribution which we attribute to spatially localized networks of coalesced Pt-nanoparticles.

## Results and discussion

### DC conductivity of nano-granular Pt/C

The intra-grain conductivity of a nano-granular metal is exceeding the inter-grain conductivity by far. This leads to the conductivity of the material being dominated by the inter-grain processes, i.e. thermally-assisted tunneling of electrons between the grains. Due to the small grain size of about 2 nm for the Pt/C system^15^, single tunnel events are associated with a Coulomb charging energy. This causes a hard transport energy gap in perfectly ordered nano-granular systems and leads to an Arrhenius-like temperature dependence of the conductivity. Beloborodov et al.^3^ showed that sequential co-tunneling over many grains, elastic at low and inelastic at higher temperatures, in combination with a random potential caused by charged impurities in the sample will soften the energy gap leading to a stretched exponential temperature dependence of the form

4 $$\begin{aligned} \sigma (T) = \sigma _0\exp \left( -\sqrt{T_0/T}\right) . \end{aligned}$$

This temperature dependence was also found for nano-granular Pt/C^13^ and is often referred to as correlated Variable Range Hopping (cVRH). The inter-grain tunnel coupling strength (normalized to the conductance quantum) denoted as *g* can be used to distinguish this insulating behavior ($g \ll 1$) from a quasi-metallic behavior ($g \ge 1$) for which the low-temperature conductance properties can be described as a granular Fermi liquid, see^14,16^. At higher temperatures and for intermediate coupling strength nano-granular metals show a logarithmic temperature dependence of the conductivity^14,17^.

**Figure 1:**
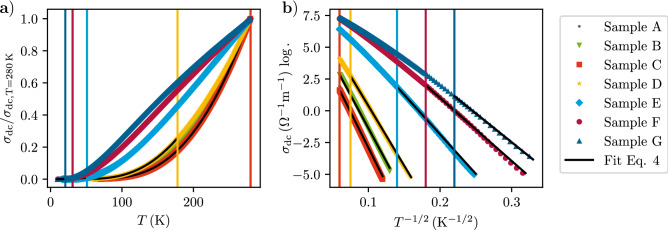
(**a**) DC conductivities of samples A to G normalized to $\sigma _{\mathrm {DC}}$ at $T=\ {280}\,\hbox {K}$. Vertical lines correspond to the upper limit of the validity range of the cVRH-behavior. (**b**) DC conductivities of the samples in $\log \sigma _{\mathrm {DC}}$ vs. $T^{-1/2}$ representation making the temperature range of validity for cVRH-behavior apparent. Fit results are presented in Table 1.

In Fig. 1 we present temperature-dependent DC conductivity data for the sample set (denoted as sample A, with smallest, to G, with largest coupling strength) from which the respective coupling strength-dependent validity ranges of cVRH-behavior can be extracted, as well as the parameters $\sigma _0$ and $T_0$ of Eq. (4) which are collected in Table 1. With this sample set we focus on the weak to intermediate coupling regime, i.e. the post-growth irradiation doses are chosen such that all samples exhibited cVRH-behavior at least at low temperatures, so that they can all be attributed to the insulating side of the insulator-metal transition of Pt/C. Equipped with these important DC conductivity reference data we now turn to the results of the AC conductivity measurements.

**Table 1:** Overview of selected parameters characterizing the set of Pt/C samples with increasing tunnel-coupling strength from sample A to G, tuned by the corresponding post-growth irradiation dose *d*.

Sample	Dose *d* ($\hbox {nC}\upmu \hbox {m}^{-2}$)	cVRH—fit with Eq. (4)		$f^*$—fit with Eq. (5)	
		$\sigma _0$ ($\Omega ^{-1}\hbox {m}^{-1}$)	$T_0$ (K)	$f_0$ ($\hbox {Hz}^{-1}$)	$T'_0$ (K)
A	0.98	$9.49\times 10^{3}$	$1.45\times 10^{4}$	$3.79\times 10^{7}$	$1.47\times 10^{4}$
B	2.95	$1.23\times 10^{4}$	$1.19\times 10^{4}$	$4.03\times 10^{7}$	$1.16\times 10^{4}$
C	4.91	$5.77\times 10^{3}$	$1.37\times 10^{4}$	$6.38\times 10^{7}$	$1.35\times 10^{4}$
D	9.82	$2.14\times 10^{4}$	$9.25\times 10^{3}$	$9.06\times 10^{7}$	$8.36\times 10^{3}$
E	29.46	$5.75\times 10^{4}$	$4.17\times 10^{3}$	$1.80\times 10^{8}$	$4.29\times 10^{3}$
F	49.09	$8.40\times 10^{4}$	$2.66\times 10^{3}$	$4.47\times 10^{7}$	$2.92\times 10^{3}$
G	98.19	$7.63\times 10^{4}$	$2.10\times 10^{3}$	$1.91\times 10^{8}$	$1.75\times 10^{3}$

Parameters $\sigma _0$ and $T_0$ are extracted from the temperature-dependent DC conductance of the samples. Parameters $f_0$ and $T_0'$ follow from the analysis of the imaginary part of the impedance $Z''(f)$. See text for details.

### AC conductivity of nano-granular Pt/C

**Figure 2:**
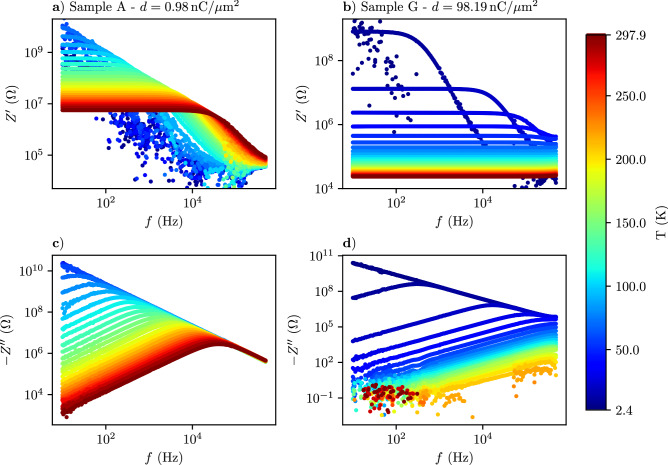
Frequency-dependence of real (resistance) and imaginary part (reactance) of impedance of selected Pt/C samples. (**a**) Real part and (**c**) imaginary part of impedance *Z* for sample A vs. frequency for various temperatures as indicated by the color code given in the legend. (**b**) Real and (**d**) imaginary part of *Z* for sample G.

In Fig. 2 we present results of the resistance $Z'$ and reactance $Z''$ vs. frequency measurements of samples A and G for various temperatures, as indicated. These data are representative for the sample set as they reflect the behavior of the nano-granular Pt/C samples from weak to intermediate tunnel coupling strength *g*. We can make the following observations. (1) At a characteristic frequency $f^*(T, g)$ the reactance exhibits a maximum which shifts to higher frequency with increasing temperature and tunnel coupling strength. (2) Correspondingly, the resistance shows a frequency-independent behavior up to the characteristic frequency above which the resistance drops, following a power law behavior before starting to saturate. (3) For sample A (weak coupling limit) the characteristic frequency can be followed up to the highest temperature in the experiment (298 K) but shifts to below the frequency range (here, 10 Hz–500 kHz). For sample G (intermediate coupling) the maximum in $Z''(f)$ is already shifted to above the available frequency range for most of the temperatures and is only visible for temperatures below about 30 K.

**Figure 3:**
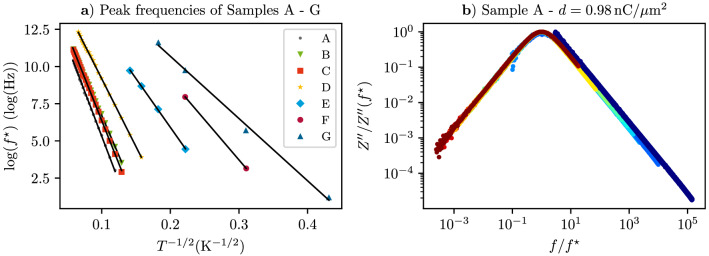
(**a**) Temperature-dependence of characteristic frequency $f^*$ in $\log {f^*}$ vs. $1/\sqrt{T}$ representation for all samples, as indicated. The solid lines are fits to the data according to Eq. (5). (**b**) Scaled representation of reactance of sample A. See text for details.

To gain further insight into this behavior we plot the characteristic frequencies vs. temperature for all samples in such a way that a comparison to the reference DC conductivity vs. temperature data can be made. This is shown in Fig. 3a. As is quite apparent from this figure, the characteristic frequency follows the same temperature dependence as the DC conductivity and can be described by

5 $$\begin{aligned} f^* = f_0 \exp \left( -\sqrt{T'_0/T}\right) . \end{aligned}$$

Fits of the data using Eq. (5) appear as solid lines in the figure. The fit parameters are collected for comparison with the fit parameters for the DC conductivity in Table 1. Apparently, the respective activation temperatures $T_0$ and $T_0'$ show a very good correspondence for all samples. In addition, $Z''(f)$ shows scaling behavior which can be seen by plotting $Z''(f)/Z''(f^*)$ vs. $f/f^*$, as is exemplarily shown in Fig. 3b for sample A.

**Figure 4:**
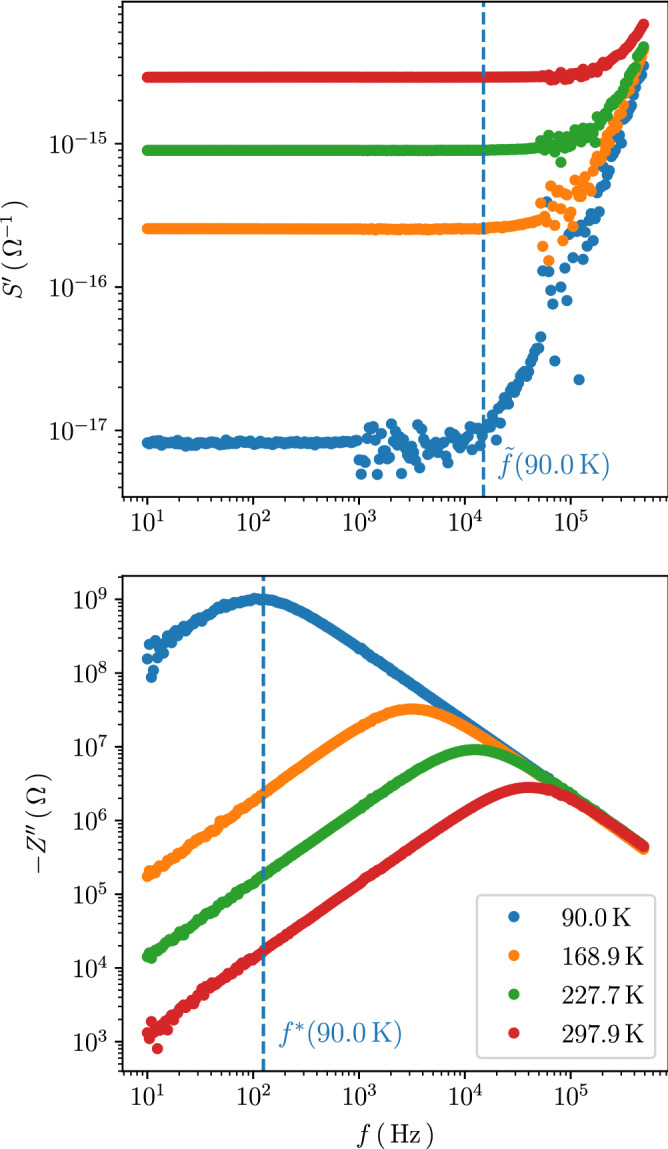
Top: Real part of frequency-dependent conductivity of sample A at temperatures as indicated. Bottom: Corresponding imaginary part of impedance vs. frequency. See text for details.

A similar behavior was also observed for nano-granular Pd/$\hbox {ZrO}_2$ in the very weak coupling limit in which the frequency-dependent conductivity was dominated by the polarization contributions^5^. However, an important difference has to be noted when comparing the Pd/$\hbox {ZrO}_2$ and Pt/C results. For Pd/$\hbox {ZrO}_2$, i.e. the very weak coupling limit, the observed scaling behavior of the frequency-dependent real part $\sigma '(f)$ of $\sigma (f)$ was related to the Barton–Nakajima–Namikawa (BNN)-relation^18–20^

6 $$\begin{aligned} \sigma _{\mathrm {DC}} = p \varepsilon _0\Delta \varepsilon \omega ^*, \end{aligned}$$

with $\Delta \varepsilon = \varepsilon (0) - \varepsilon _\infty$ being the difference between the low and high frequency value of the dielectric function, $\varepsilon _0$ the vacuum permittivity, $\omega ^* = 2\pi f^*$ and *p* a constant of order one^21–23^. Since $\Delta \varepsilon$ is generally less temperature-dependent than $\sigma _{\mathrm {DC}}$ and $\omega ^*$, the observed proportionality $\sigma _{\mathrm {DC}} \propto \omega ^*$ can be justified^1^. For Pt/C however, a scaling of $\sigma '$ of the form

7 $$\frac{\sigma '(f)}{\sigma _{\mathrm {DC}}} = F\left( \frac{f}{f^*}\right) .$$

by using $f^*$ as extracted from the maximum of $Z''(f)$ does not succeed. The reason for this becomes apparent when plotting $\sigma '$ vs. *f* and comparing the characteristic frequency ${\tilde{f}}$ above which $\sigma '(f)$ starts to deviate from its frequency-independent behavior and goes over into a power-law behavior with exponent larger than 2. This is shown exemplarily for sample A in Fig. 4a. By comparing ${\tilde{f}}$ to the corresponding characteristic frequency $f^*$ extracted from $Z''(f)$ the difference becomes apparent. The maximum in $Z''(f)$ occurs at a frequency where $\sigma '(f)$ is still frequency-independent. In the next sub-section we discuss the reasons for the observed behavior in Pt/C within a lumped circuit analysis.

### Lumped circuit analysis of AC conductivity

**Figure 5:**
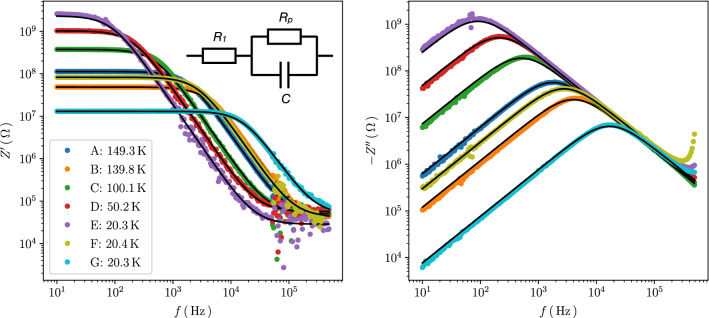
Frequency dependencies of real (left) and imaginary parts (right) of impedance for all samples at selected temperatures as indicated. The black lines are results of a lumped circuit fit; see inset for circuit model. See text for details.

The reasons for the scaling behavior of $Z''(f)$ discussed in the previous section become apparent when doing a lumped circuit analysis. Based on the simple model circuit depicted in the inset of Fig. 5 (left) the frequency dependence of $Z'$ and $Z''$ can be reproduced quantitatively. The rationale for the model circuit is as follows. First, considering the electrode-sample setup depicted in the method section (see Fig. 7) a serial resistance stemming from the interface region of the Au/Cr electrodes to the Pt/C nano-granular material can be expected. In fact, an additional parallel capacitance has to be assumed. The simulations do however show that this capacitance would be too small to be visible in the data within the frequency range available to us. Second, for any disordered granular metal coalescence of metallic grains will statistically happen in some regions of the sample and this will be reflected as another serial resistance contribution. The model circuit resistor denoted $R_1$ represents both of these possible contributions. We will discuss below which we consider to be dominant. Third, the most simple model for taking the granular nature of Pt/C into account is that of a parallel circuit of a resistor and a capacitance which reflects the tunneling nature of the charge transport inside Pt/C. This is taken into account by the parallel circuit of $R_p$ and *C*.

In Fig. 5 we show a subset of all spectra that we measured covering all samples. We chose for each sample a temperature for which the low-pass behavior, exhibited in the spectra of all samples, does fully encompass the frequency-independent part of $Z'$ at low frequencies and a saturation behavior at higher frequencies (see “Supplementary material” for a full account on all spectra). This allows us to extract the parameters $R_1$ and $R_p + R_1 \equiv R_0$ directly from the spectra, so that only the capacitance parameter *C* has to optimized in the non-linear curve fit. It is quite apparent that both, $Z'(f)$ and $Z''(f)$, can be nicely fitted by the simple model circuit.

**Figure 6:**
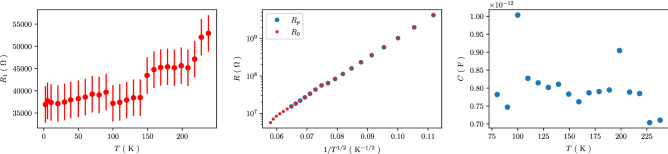
Temperature dependence of lumped circuit fit parameters for sample A. $R_1$ and $R_0$ are taken directly from the frequency dependence of the real part of the impedance. *C* is a fit parameter following the lumped circuit model, as detailed in the text. $R_p = R_0 - R_1$. The error bars for $R_1$ correspond to the standard deviation of $R_1$ taken from the real part of the impedance at 80 K for $f \ge 0.2\,$MHz where saturated behavior is clearly observed.

In Fig. 6 we compile the parameters $R_1(T)$, $R_0(T)$, $R_p(T)$ and *C*(*T*) for sample A, representing the general behavior we observed from the lumped circuit fit for all samples. The main observations are that (a) the serial resistor $R_1$ tends to drop with decreasing temperature, (b) the resistor $R_P$ shows c-VRH behavior over a wide temperature range and (c) the capacitance *C* is temperature-independent, albeit with some scatter around a mean value of about $0.8\,$pF. We note that the magnitude of $R_1$ is orders of magnitude smaller than the magnitude of $R_p$ for all temperatures, which is why the temperature-dependent values of $R_p$ and $R_0$ differ so little that they cannot be discriminated in the plot. The temperature dependence of $R_1$ indicates metallic behavior which may be expected from metallic regions inside the Pt/C sample with coalesced Pt grains. A transfer resistance from Cr/Au to Pt/C would be expected to increase with decreasing temperature, as it is known that the metal volume fraction in Pt/C from FEBID is reduced in the initial growth stages, i.e. close to the interface at the Cr/Au electrodes. This argument does, of course, rely on the additional assumption that the transfer resistance—which must be there—is significantly smaller than the non-percolating resistance contribution from the coalesced Pt grains. Our argument about the actual origin of $R_1$ therefore has to remain speculative in nature.

Based on the results of the lumped circuit analysis we conclude that the observed scaling behavior of $Z''(f)$ discussed in the previous section is due to the fact that the temperature dependence of $R_p$ is just like that of the DC resistance of the samples. Consequently, scaling will occur if the other parameters, $R_1$ and *C*, do either show no appreciable temperature dependence (*C*) or are just negligible as compared to $R_p$ ($R_1$).

## Conclusion

The AC conductivity of nano-granular Pt/C on the insulating side but close to the insulator-metal transition is dominated by the free charges contribution to the conductivity, i.e. by the tunneling of the charge carriers. The correlated electron transport, as expressed by cVRH at low temperatures and depending on the tunnel coupling strength, is reflected in the effective lumped-circuit resistance $R_p$ which coincides with the DC resistance of the samples. It is found that a single temperature-independent capacitance *C* in the lumped-circuit analysis is sufficient to describe the frequency-dependent impedance for all samples and over the full temperature range. These observations are in contradistinction to previous work on nano-granular Pd/$\hbox {ZrO}_2$ in the very weak-coupling regime where the polarization part of the AC conductivity dominates, a bimodal particle size distribution can be deduced from the spectra, and a universal behavior of the real part of the conductivity was found^4,5^. From both, the universal conductance behavior in the very weak-coupling regime (Pd/$\hbox {ZrO}_2$) and the tunnel-coupling strength independent general low-pass behavior close to the insulator-metal transition (Pt/C), a potentially interesting application of nano-granular metals in frequency-dependent dielectric sensing can be anticipated. In previous work we could show that the DC conductivity of thin nano-granular Pt/C layers can act as sensitive probes of changes in the dielectric properties in the medium close to the nano-granular metal^9^. This was used to follow the paraelectric-to-ferroelectric phase transition in individual islands of an organic ferroelectric^8^ and in detecting the presence of (sub-)monolayers of water for relative humidity sensing^24^. Taking advantage of the frequency dependence of the AC conductivity of nano-granular metals a new type of impedance spectroscopy can be envisioned. This would be based on the dielectric changes of layers in close proximity to a nano-granular sensing element or changes of the dielectric properties of the insulating matrix of the nano-granular metal itself which would be detectable by AC conductance changes of the nano-granular metal. In particular in combination with FEBID highly-miniaturized dielectric sensor elements could be realized. As a promising example we envision the combination of Pt/C with surface-anchored metal-organic frameworks (MOF) which can act as hosts for guest molecules whose presence will locally modify the MOF’s dielectric properties and thus lead to changes in the nano-granular metal’s AC conductance. The demonstration of this new sensing approach, for which the notion proximity impedance spectroscopy may be used, has to remain for future exploration.

## Methods

The focused electron beam induced deposition (FEBID) process using the precursor ($(\hbox {CH}_{3})_{3}\hbox {CH}_{3}\hbox {C}_{5}\hbox {H}_{4}\hbox {Pt}$) was performed in an FEI Nova NanoLab 600 dual-beam electron- and ion-beam microscope. The base pressure of the system was $3.5\times 10^{-7}\,\hbox {mbar}$. The precursor was heated inside a gas injection system to ${44}^\circ \hbox {C}$ and introduced into the vacuum chamber with an injection needle of 0.5 mm inner diameter positioned about 0.1 mm above the substrate surface which led to a deposition pressure of approx. $5.0\times 10^{-6}\,\hbox {mbar}$. The beam was set to $5\,\hbox {kV}$ acceleration voltage carrying a current of $1.6\,\hbox {nA}$ and rastered in an $8\,\upmu \hbox {m}\times 1\,\upmu \hbox {m}$ area between the gold electrodes. The area was covered with a point pitch of 20 nm, a dwell time at each point of $1\,\upmu \hbox {s}$ in a serpentine fashion with 2000 repetitions (passes) to reach an overall deposition dose of $7.85\,\hbox {nC}\upmu \hbox {m}^{-2}$. These parameters where used to reach a deposit height in the as deposited state, as measured *in situ* with a GETec Microscopy AFSEM atomic force microscope (AFM), of approx. $100\,\hbox {nm}$. After growth the seven samples (A–G) were exposed to a post-growth irradiation step using the same beam parameters as during the deposition process but with different passes. This resulted in irradiation doses ranging from $1\,\hbox {nC}\upmu \hbox {m}^{-2}$ (A) to $100\,\hbox {nC}\upmu \hbox {m}^{-2}$ (G). During the irradiation process, which increases the inter-granular tunnel coupling by modification of the carbon matrix^13,15^, the DC-conductivity of the samples was monitored in situ. The heights of the samples after the post-growth treatment were measured again using a nanosurf Easyscan 2 AFM in air and found to be reduced by about 20–40%, depending on the irradiation dose, as compared to the heights of the as-grown samples. This is a known consequence of the irradiation treatment which leads to electron-induced desorption of residual volatile precursor fragments and an increase of the sample density^15^. These heights were used to calculate the conductance-to-conductivity conversion factors for the samples.

**Figure 7:**
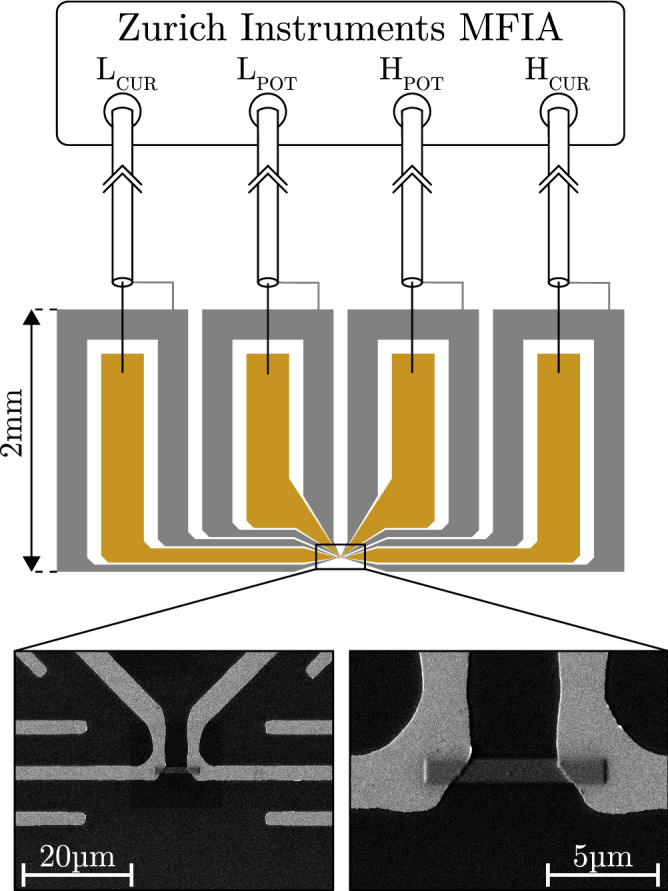
Measurement setup with electrode layout and an example of a Pt/C FEBID deposit. SEM images of a deposit between gold electrodes on an $\hbox {Al}_{2}\hbox {O}_{3}$-substrate. Additional electrodes, shown in grey, reduce capacitive coupling between the signal electrodes and are connected to the shields of the cryostat’s coaxial cables, which are then connected to the measurement device.

The temperature-dependent conductance measurements were performed inside an Oxford Instruments $^4$He cryostat with a variable temperature insert in the range of 2–300 K. The temperature dependent DC-conductance was recorded using a Keithley Instruments 2636A sourcemeter. For the AC-conductance measurements a Zurich Instruments MFIA impedance analyzer was used performing frequency-dependent measurements between 10 Hz and 5 MHz at fixed temperatures with an accuracy of $\pm 0.1\hbox { K}$ in a four-terminal set-up with 0.1 V signal amplitude. As depicted in Fig. 7 the samples where deposited in a rectangular manner in between short-circuited pairs of the four electrodes provided on a $\hbox {Al}_{2}\hbox {O}_{3}$-substrate. The electrode structure was defined by means of UV-lithography and DC-magnetron sputtering of a 45 nm gold layer with an adhesive layer of 5 nm chromium in a lift-off process. The used electrode design was the result of several electrode iterations and associated AC-conductance measurements combined with equivalent circuit calculations in order to identify the electrode setup with the least capacitive coupling between the individual electrodes. In the final design the two high and low terminal pairs were short-circuited, respectively. Furthermore, additional planar shield electrodes were introduced on the substrate and connected to the outer conductor of the cryostat’s coaxial-cables as shown in Fig. 7. The gold electrodes on the substrate were gold wire-bonded to gold pins on a printed circuit board sample mount to which the stainless-steel coaxial-cables of the cryostat were soldered and finally connected to the measurement device. Further parasitic effects of the cables were reduced employing a Short-Open-Compensation using samples prepared according to the aforementioned UV-lithography process.

## Supplementary information

Supplementary Information.
