# Supplementary material for: AC conductivity and correlation effects in nano-granular Pt/C
Source: Sci Rep. 2021 Jul 26;11:15163. doi: 10.1038/s41598-021-94575-w (PMC8313567; doi:10.1038/s41598-021-94575-w)
Supplement: Supplementary file 1 — Supplementary Information. [file 41598_2021_94575_MOESM1_ESM.pdf]

Supplementary Information (SI)
AC Conductivity and Correlation Effects in Nano-Granular Pt/C

Marc Hanefeld[1], Peter Gruszka[1], and Michael Huth[1,*]

[1]Physikalisches Institut, Goethe Universität, Frankfurt am Main, D-60438, Germany
[*]michael.huth@physik.uni-frankfurt.de

ABSTRACT

Full Set of Impedance Spectra and their Lumped-Circuit Analysis

General Remarks

In this supplementary information we present the impedance spectra of all samples (A to G) taken at 10 K intervals from about 2 K to about 298 K. We used the lumped-circuit model as described in the main text to fit the spectra at selected temperatures for each sample. The temperatures selected where those for which the low-pass behavior did fully encompass the frequency-independent part of Z' at low frequencies and a saturation behavior at higher frequencies. This allowed us to extract the parameters R_1 and $R_p + R_1 \equiv R_0$ directly from the spectra, so that only the capacitance parameter C had to optimized in the non-linear curve fit. The results of this fit are also shown as black solid lines in the spectra. The temperature dependence of the parameters R_1, R_p (R_0) and C are shown in separate figures (see below).

Careful analysis of the spectra reveals for all samples and for some temperatures deviations in $Z'(f)$ from the otherwise consistently observed low-pass behavior. These deviations manifest themselves as the occurrence of small negative values for $Z'(f)$ in a limited frequency range above the onset frequency for the low-pass behavior; see data taken at 99.9 K in Fig. 1 for an example (in the log-log representation of the spectra the negative $Z'(f)$ are, of course, omitted). We are not sure what causes these deviations but we do not consider these to be characteristic for the nano-granular metal but rather assume unstable behavior in the guard potentials caused by the impedance analyzer to be cause.

Sample A

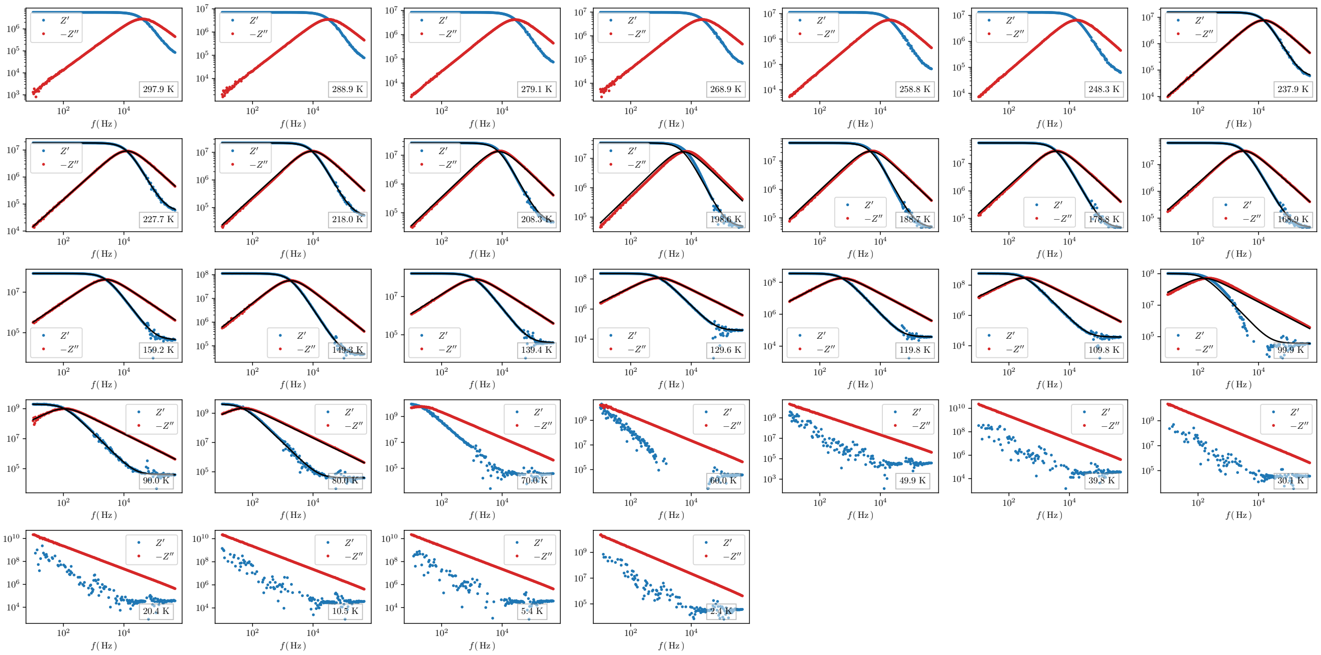

Figure 1. Overview of impedance spectra of sample A at temperatures as indicated. Black lines refer to fits according to the lumped-circuit model depicted in the inset of Fig. 5 (left) in the main text.

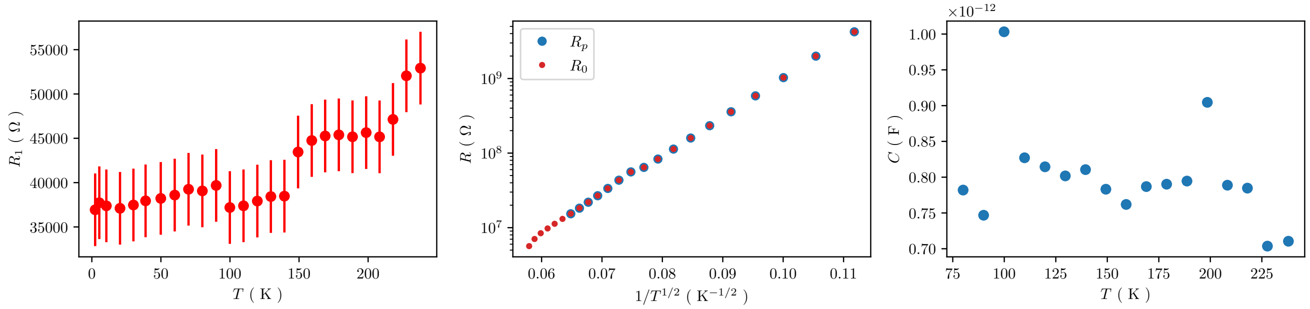

Figure 2. Temperature dependence of lumped circuit fit parameters for sample A. R_1 and R_0 are taken directly from frequency dependence of the real part of the impedance. C is a fit parameter following the lumped circuit model, as detailed in the main text. $R_p = R_0 - R_1$. The error bars for R_1 correspond to the standard deviation of R_1 taken from real part of the impedance at 80 K for $f \geq 0.2$ MHz where saturated behavior is clearly observed.

Sample B

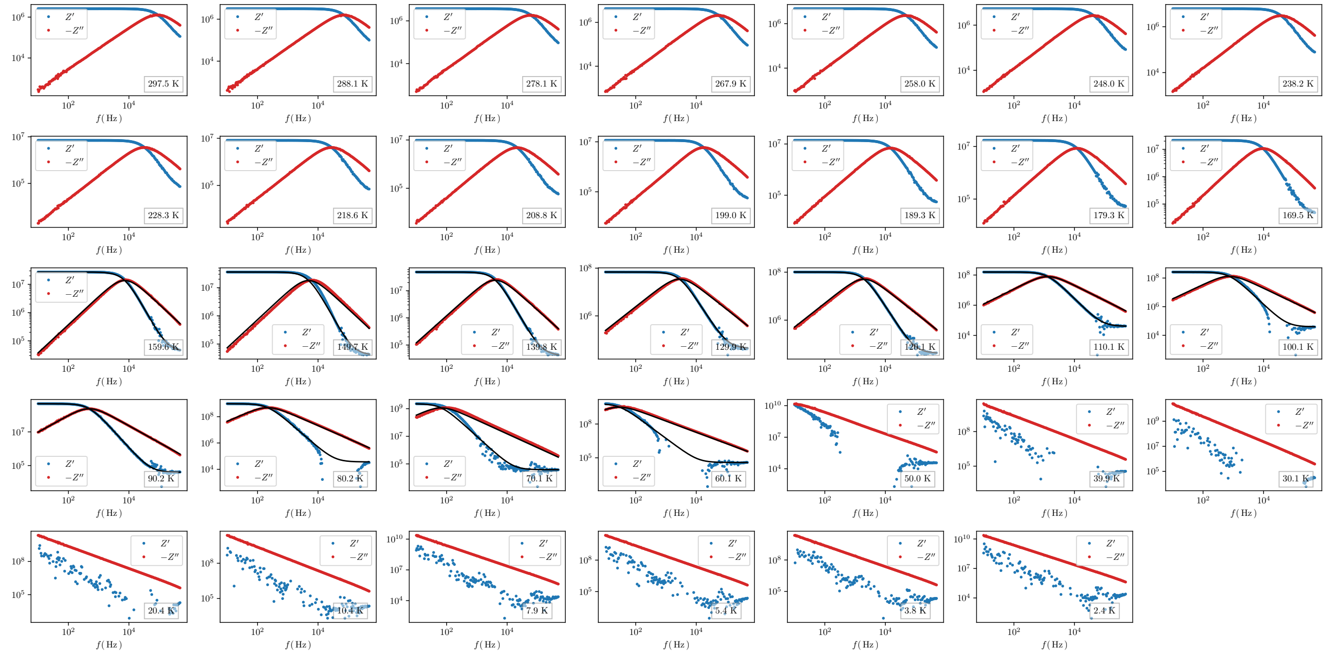

Figure 3. Overview of impedance spectra of sample B at temperatures as indicated. Black lines refer to fits according to the lumped-circuit model depicted in the inset of Fig. 5 (left) in the main text.

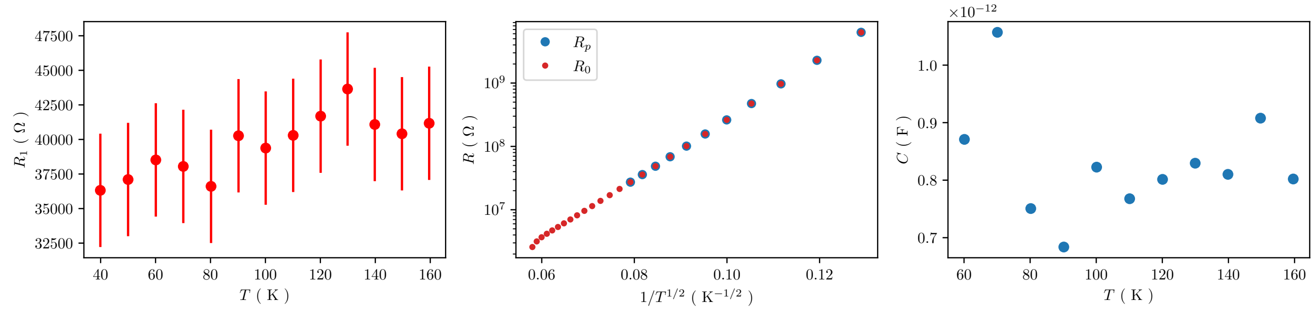

Figure 4. Temperature dependence of lumped circuit fit parameters for sample B. R_1 and R_0 are taken directly from frequency dependence of the real part of the impedance. C is a fit parameter following the lumped circuit model, as detailed in the main text. $R_p = R_0 - R_1$. The error bars for R_1 correspond to the standard deviation of R_1 taken from real part of the impedance at 70 K for $f \geq 0.1$ MHz where saturated behavior is clearly observed.

Sample C

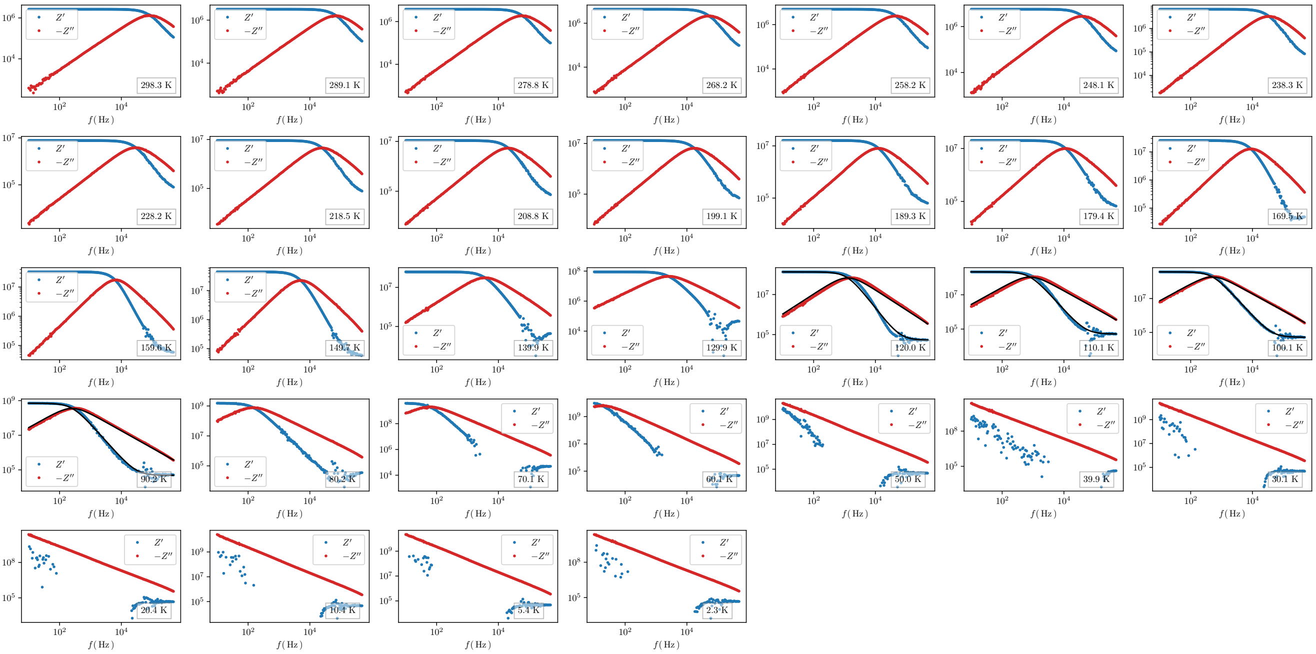

Figure 5. Overview of impedance spectra of sample C at temperatures as indicated. Black lines refer to fits according to the lumped-circuit model depicted in the inset of Fig. 5 (left) in the main text.

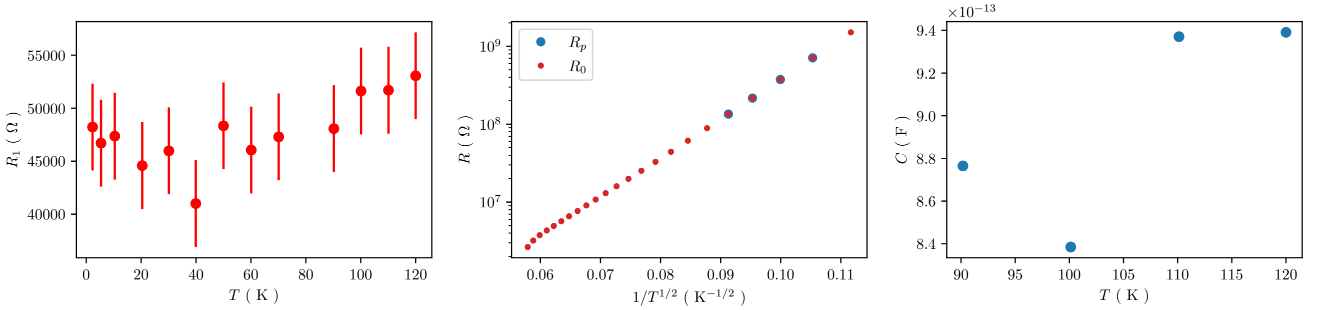

Figure 6. Temperature dependence of lumped circuit fit parameters for sample C. R_1 and R_0 are taken directly from frequency dependence of the real part of the impedance. C is a fit parameter following the lumped circuit model, as detailed in the main text. $R_p = R_0 - R_1$. The error bars for R_1 correspond to the standard deviation of R_1 taken from real part of the impedance at 50 K for $f \geq 0.2$ MHz where saturated behavior is clearly observed.

Sample D

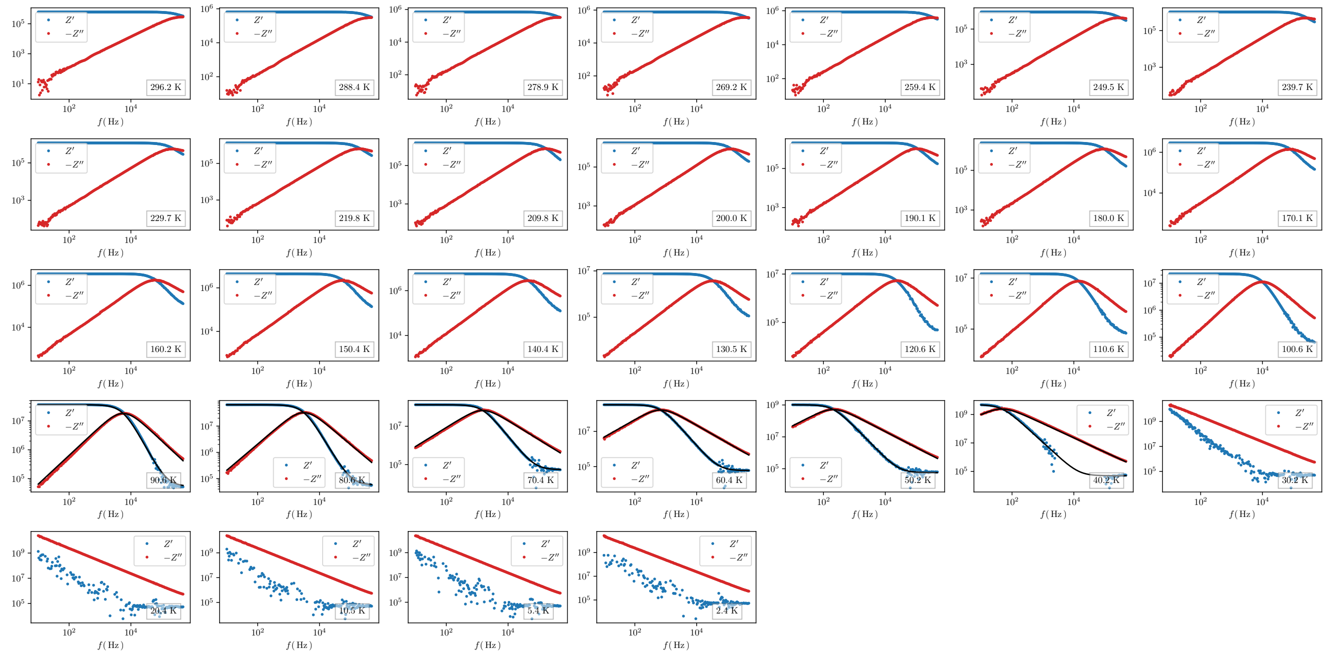

Figure 7. Overview of impedance spectra of sample D at temperatures as indicated. Black lines refer to fits according to the lumped-circuit model depicted in the inset of Fig. 5 (left) in the main text.

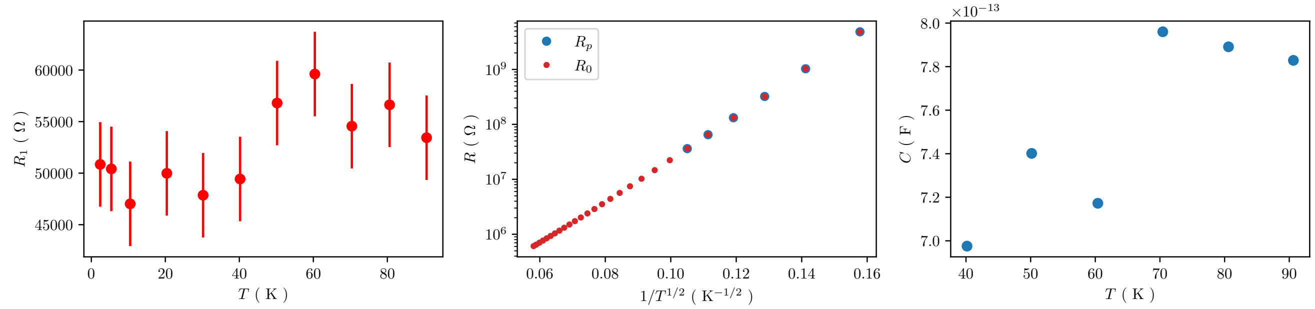

Figure 8. Temperature dependence of lumped circuit fit parameters for sample D. R_1 and R_0 are taken directly from frequency dependence of the real part of the impedance. C is a fit parameter following the lumped circuit model, as detailed in the main text. $R_p = R_0 - R_1$. The error bars for R_1 correspond to the standard deviation of R_1 taken from real part of the impedance at 50 K for $f \geq 0.2$ MHz where saturated behavior is clearly observed.

Sample E

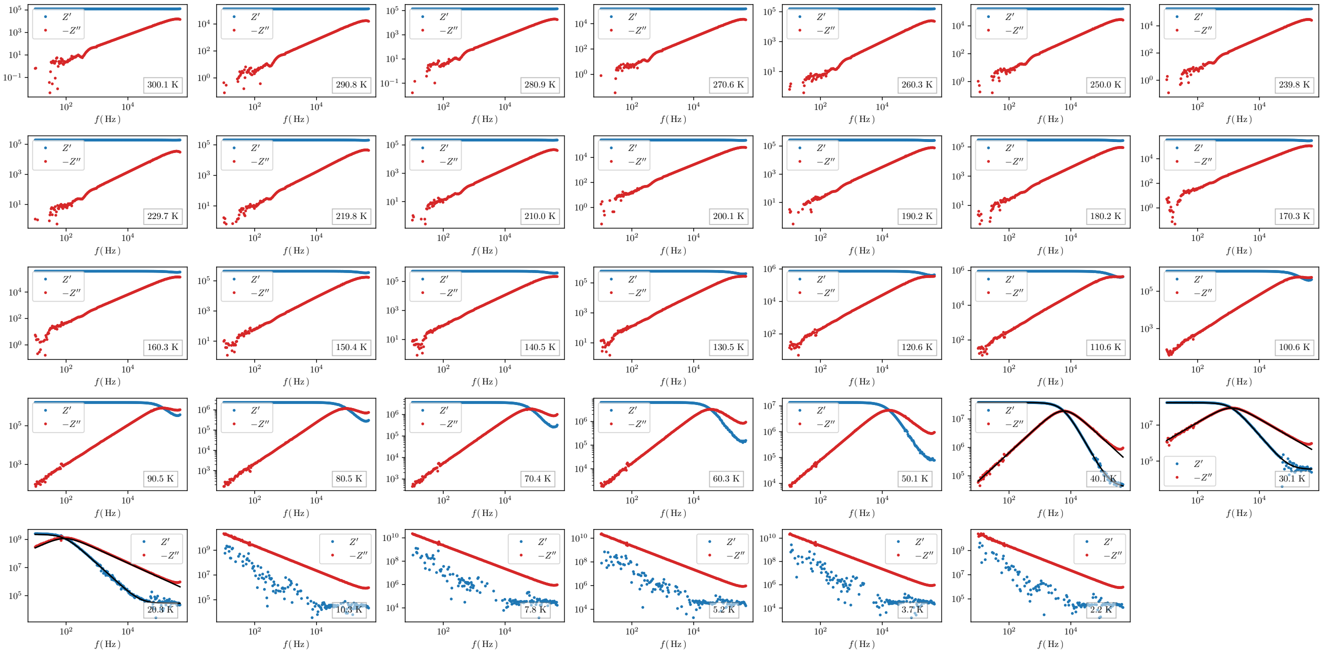

Figure 9. Overview of impedance spectra of sample E at temperatures as indicated. Black lines refer to fits according to the lumped-circuit model depicted in the inset of Fig. 5 (left) in the main text.

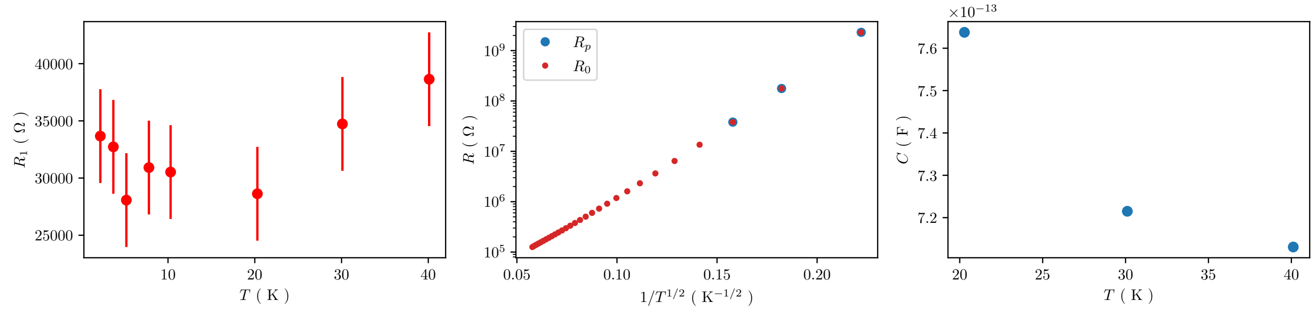

Figure 10. Temperature dependence of lumped circuit fit parameters for sample E. R_1 and R_0 are taken directly from frequency dependence of the real part of the impedance. C is a fit parameter following the lumped circuit model, as detailed in the main text. $R_p = R_0 - R_1$. The error bars for R_1 correspond to the standard deviation of R_1 taken from real part of the impedance at 20 K for $f \geq 0.2$ MHz where saturated behavior is clearly observed.

Sample F

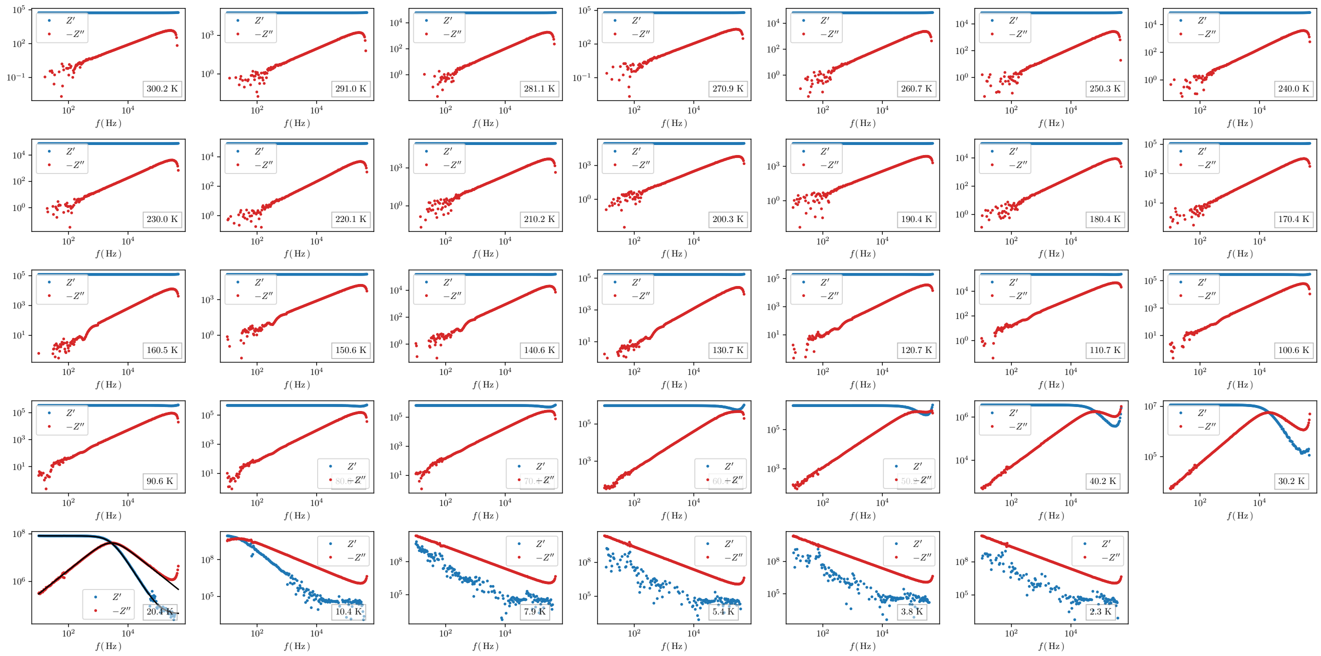

Figure 11. Overview of impedance spectra of sample F at temperatures as indicated. Black lines refer to fits according to the lumped-circuit model depicted in the inset of Fig. 5 (left) in the main text.

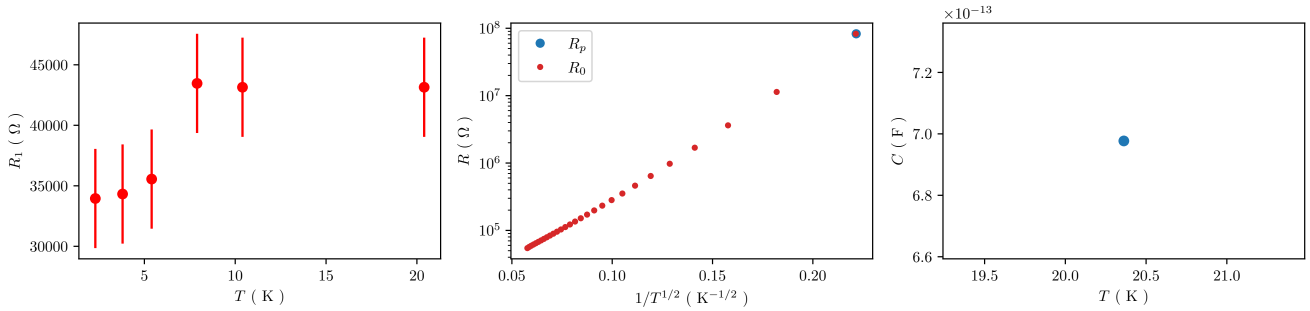

Figure 12. Temperature dependence of lumped circuit fit parameters for sample F. R_1 and R_0 are taken directly from frequency dependence of the real part of the impedance. C is a fit parameter following the lumped circuit model, as detailed in the main text. $R_p = R_0 - R_1$. The error bars for R_1 correspond to the standard deviation of R_1 taken from real part of the impedance at 10 K for $f \geq 0.1$ MHz where saturated behavior is clearly observed.

Sample G

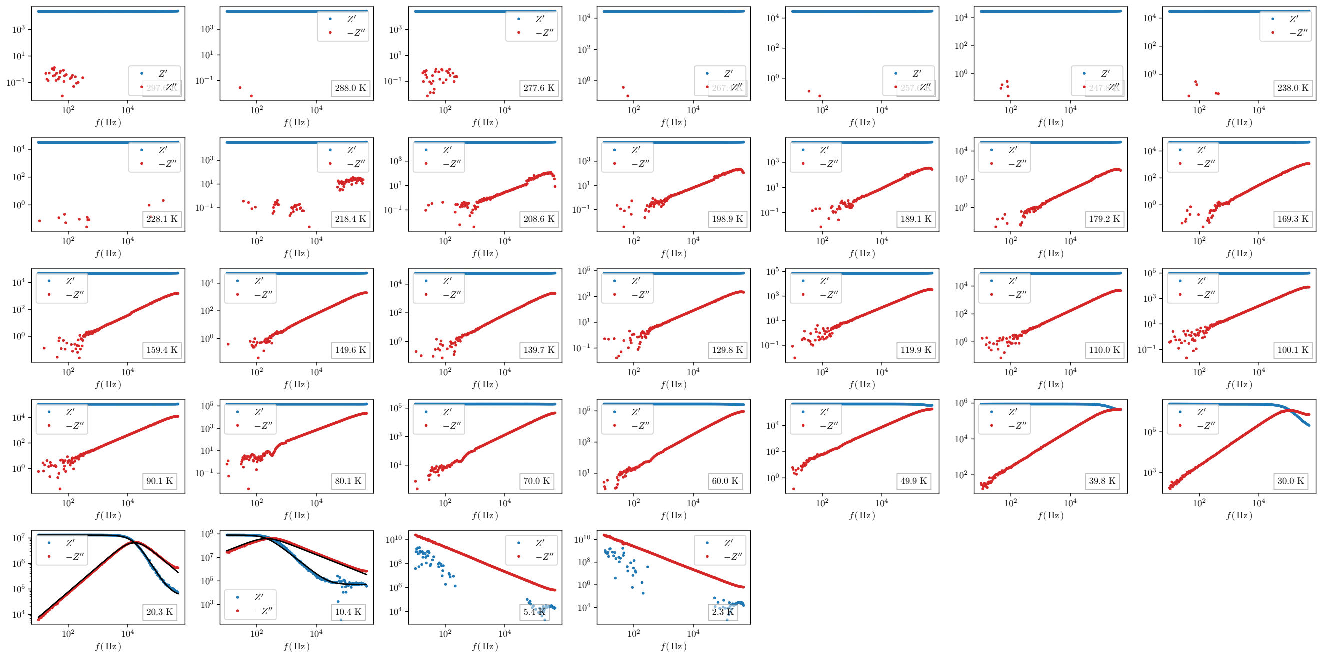

Figure 13. Overview of impedance spectra of sample G at temperatures as indicated. Black lines refer to fits according to the lumped-circuit model depicted in the inset of Fig. 5 (left) in the main text.

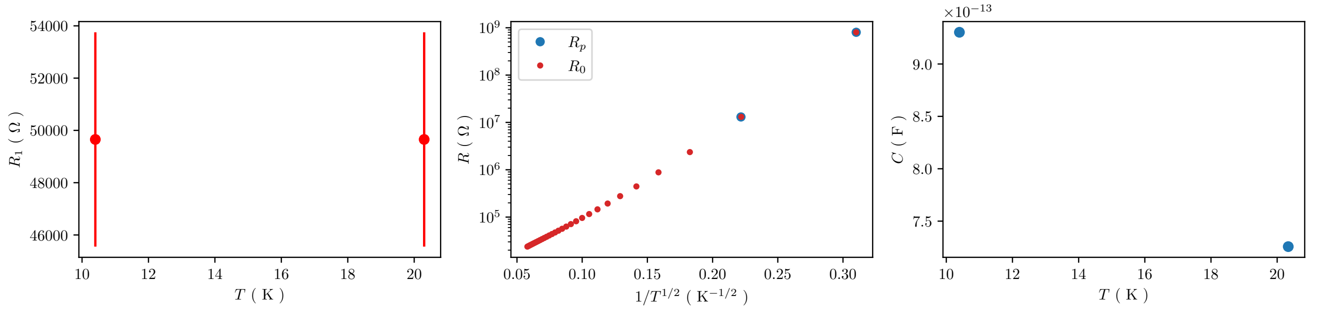

Figure 14. Temperature dependence of lumped circuit fit parameters for sample G. R_1 and R_0 are taken directly from frequency dependence of the real part of the impedance. C is a fit parameter following the lumped circuit model, as detailed in the main text. $R_p = R_0 - R_1$. The error bars for R_1 correspond to the standard deviation of R_1 taken from real part of the impedance at 10 K for $f \geq 0.2$ MHz where saturated behavior is clearly observed.
